# Effect of mechanical damage and wound healing on the viscoelastic properties of stems of flax cultivars (*Linum usitatissimum* L. cv. Eden and cv. Drakkar)

**DOI:** 10.1371/journal.pone.0185958

**Published:** 2017-10-05

**Authors:** Cloé Paul-Victor, Sara Dalle Vacche, Federica Sordo, Siegfried Fink, Thomas Speck, Véronique Michaud, Olga Speck

**Affiliations:** 1 Plant Biomechanics Group and Botanic Garden, University of Freiburg, Freiburg, Germany; 2 Freiburg Materials Research Center (FMF), Freiburg, Germany; 3 Laboratory for Processing of Advanced Composites (LPAC), Institute of Materials, Ecole Polytechnique Fédérale de Lausanne (EPFL), Lausanne, Switzerland; 4 Institute for Forest Botany, University of Freiburg, Freiburg, Germany; 5 Competence Network Biomimetics, Freiburg, Germany; CNRS, France, FRANCE

## Abstract

As plant fibres are increasingly used in technical textiles and their composites, underlying principles of wound healing in living plant fibres are relevant to product quality, and provide inspiration for biomimetic healing in synthetic materials. In this work, two *Linum usitatissimum* cultivars differing in their stem mechanical properties, cv. Eden (stems resistant to lodging) and cv. Drakkar (with more flexible stems), were grown without wound or with stems previously wounded with a cut parallel or transversal to the stem. To investigate wound healing efficiency, growth traits, stem biomechanics with Dynamic Mechanical Analysis and anatomy were analysed after 25-day recovery.

Longitudinal incisions formed open wounds while transversal incisions generated stem growth restoring the whole cross-section but not the original stem organisation. In the case of transversal wound healing, all the bast fibre bundles in the perturbed area became lignified and pulled apart by parenchyma cells growth. Both *Linum* cultivars showed a healing efficiency from 79% to 95% with higher scores for transversal healing. Morphological and anatomical modifications of *Linum* were related to mechanical properties and healing ability. Alongside with an increased understanding of wound healing in plants, our results highlight their possible impact on textile quality and fibre yield.

## Introduction

From a material scientist’s point of view, stems of non-woody plants can be considered as fibre-reinforced composites, which are made up of unlignified collenchyma fibres or lignified sclerechyma fibres embedded in a matrix of lignified or unlignified parenchymatous tissues. Having in mind that the capability to repair external and internal damage at different length scales (from the molecular level up to entire organisms) is one of the key factors for survival in all life forms, it is probable that the repair of these fibre-reinforced composites has evolved independently in different lineages of higher plants. Until now, interest has been focused mainly on wound healing in trees and vegetables for commercial and agricultural purposes [1–6]. During the last few years, self-repair in plants has become a focus of material scientists as role models for the development of biomimetic self-repairing materials [7–9]. A differentiation has been made between acellular repair mechanisms such as sealing and healing of damage with the help of latex and tree resin [10], cellular sealing processes with parenchymatous repair cells as found in in woody vines [11], and repair processes at organ level such as the movement of succulent leaves until the wound edges are in close contact [12]. Wound healing mechanisms of plant fibres have yet been surprisingly little studied despite their high implications in plant life history and development.

Besides the knowledge transfer of self-repair mechanisms from biological concept generators into biomimetic technical applications, natural fibres are directly used to produce technical materials, under the form of textiles and their composites. First, environmental concerns led structural materials research towards materials that are of renewable origin, widely produced in the world and potentially biodegradable [13–15]. Reproducible quality and mechanical properties of plant fibres are thus essential for engineering materials [16, 17]. Environmental conditions during plant growth may include mechanical damage or herbivory [18] issues during their production, making the understanding of wound healing mechanisms for non-tree plant fibres necessary. Second, as the field of self-healing materials is rapidly growing [19, 20], an increased knowledge of wound healing mechanisms in plants in general and plant fibres in particular may lead to biomimetic routes to sealing and healing in technical materials.

Recently, a plant fibre distinguished itself from other non-tree fibres as a promising replacement for glass fibres as reinforcement in polymeric matrix composites: flax [17, 21–24]. Processed flax fibres are characterized by a low density, a high-aspect ratio and good mechanical properties ranging from 19 to 103 GPa for the Young's modulus and from 264 to 2000 MPa for the tensile strength [15, 16, 23]. These specificities establish the flax fibres as serious competitors to traditional man-made technical reinforcing fibres [16, 25, 26]. Flax fibres, also known as bast fibres, derive from *Linum usitatissimum* L. stems. They consist of sclerenchyma cell bundles with low lignin content located in the outer periphery of the vascular cylinder. They originate from the primary phloem, more specifically from the protophloem [27, 28].

In the present study, we investigated the wound healing mechanisms of *Linum usitatissimum* and especially of its bast fibres and therefore analysed the plants during their growth, contrary to most studies about *Linum usitatissimum* fibres conducted on processed fibres. More specifically, our aims were to determine: (1) which effects fibre damage exerts on the mechanical properties and structure of the stem (wound healing efficiency); (2) to what extent the fibres are repaired and (3) how are the fibres affected in in terms of tissue organisation and histological changes.

## Materials and methods

### Plant material and experimental design

**Fig 1:**
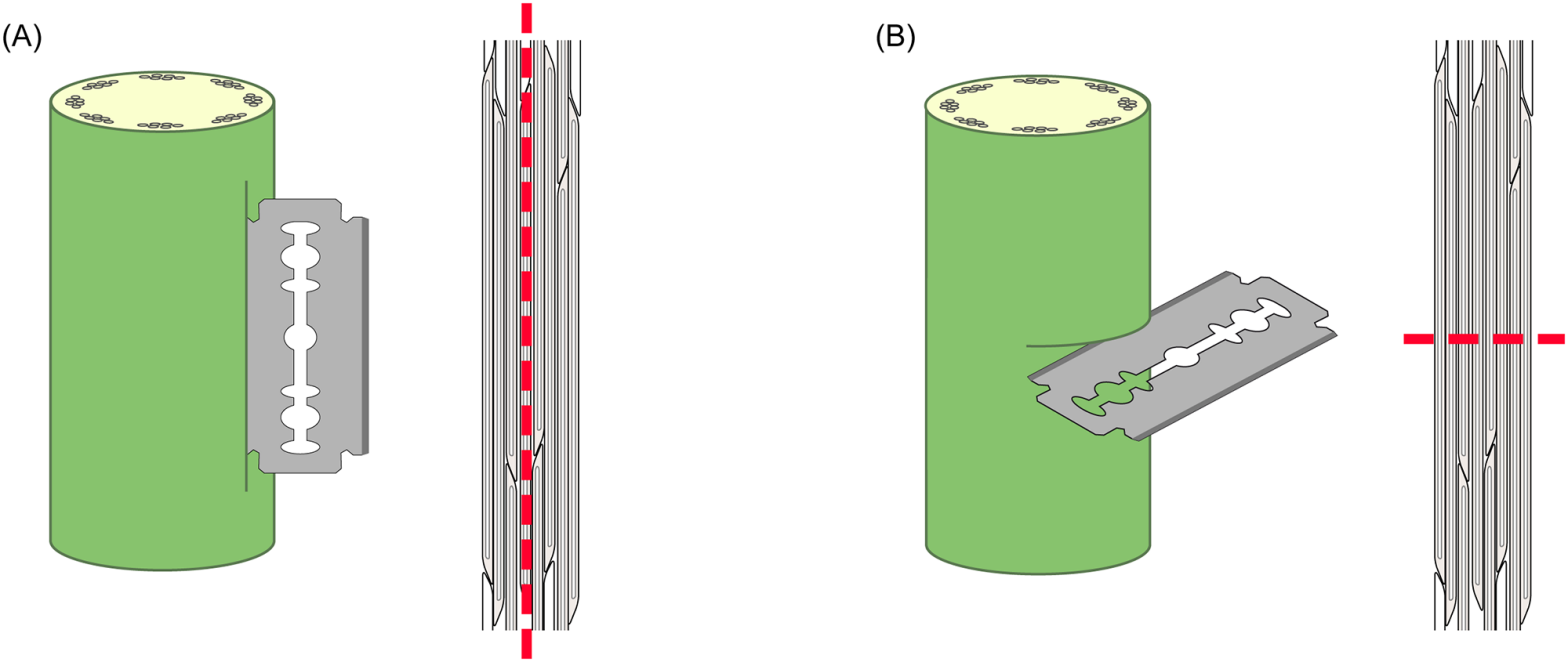
Schematic drawing of wound types. The two types of mechanical damage performed with a razor blade on *Linum* stems showing (a) longitudinal wound with a cut parallel to the fibres and (b) transversal wound with a cut perpendicular to the fibres.

Seeds were obtained from the company "Terre de Lin" in Normandy (France) specialized in *Linum usitatissimum* cultivars. Two *Linum* cultivars for textile use differing in their stem mechanical properties were selected: cv. Eden (stems resistant to lodging) and cv. Drakkar (with more flexible stems). Seeds were sown the 13th of March 2014 (0 Days After Sowing: DAS). Following germination (two cotyledons fully developed), seedlings were planted with two plants per pot and were grown in 12-cm diameter and 7-cm deep pots filled with compost. Watering regime was set-up daily to ensure non-limited water supply during growth. All plants were cultivated outside in the Botanic Garden of Freiburg, Germany. At 69 DAS, each plant was marked at 5 cm from the stem apex. A third of all the plants were kept as control without wound, a third was wounded with a cut parallel to the fibres (Fig 1A) and the last third wounded with a cut perpendicular to the fibres (Fig 1B). Wounds were performed below the 5 cm-mark with a razor blade (Derby Extra Double Edge Razor Blade) where wound depth and length were controlled with a blade holder to ensure a reproducible wound size (around 400-μm deep and 3-cm long). Depth was selected in order to damage the sclerenchyma fibres distributed in the stem periphery. Plants were then left to recover until the mechanical measurement at 95 DAS.

### Measurement of morphological traits and mechanical properties

**Fig 2:**
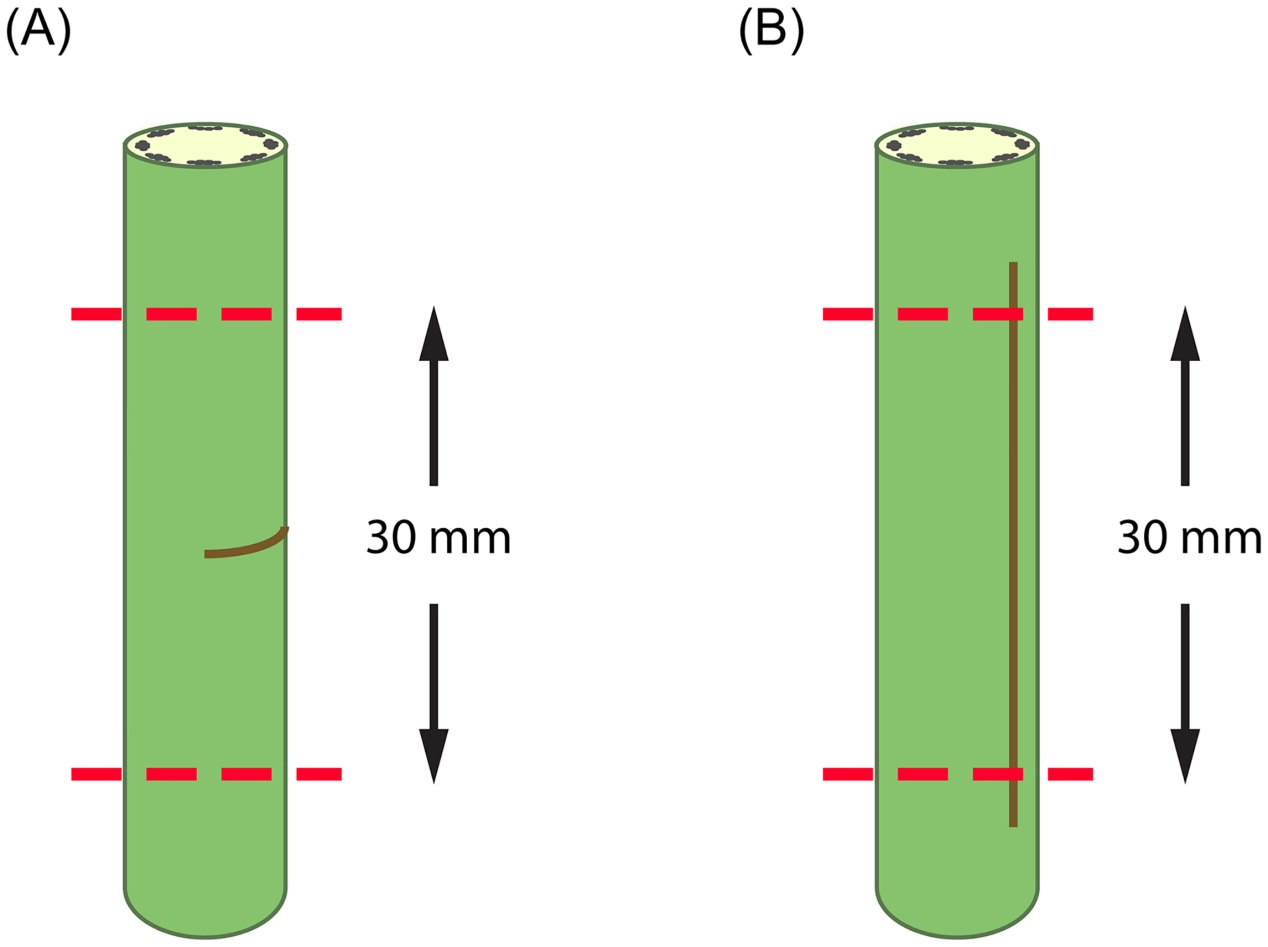
Schematic drawing of the sampling area in relation to the wound in order to perform the DMA tests. (A) sampling area of a transversal wounded stem and (B) sampling area of a longitudinal wounded stem.

Plant height was measured prior to mechanical tests at 95 DAS (just before flowering stage). 3-cm long segments of *Linum* stems were taken from below the mark previously made at 69 DAS containing either no wound for the controls, a longitudinal wound for parallel damage to the fibres or a transversal wound for damage perpendicular to the fibres (Fig 1). The maximum sample length fitting the DMA test was 30 mm, therefore samples of 30 mm were used to perform the mechanical analysis. The resulting length between the clamps was 13–16 mm. In the case of a transversal wound, the wound was placed in the middle and the sample cut 15 mm below and above the wound to obtain 30 mm segment (Fig 2A). In the case of a longitudinal wound, the middle of the wound was used as reference point and the sample cut 15 mm below and above this point to obtain 30 mm segment (Fig 2B). Diameters were measured at four points (every 1 cm) along each segment and along two perpendicular directions. In the case of a wounded segment, one of the directions was along the wound. The cross-sectional area was calculated on basis of the measured diameters, whereby the control plants have an almost perfect round shape. The healed cross-sections have an elliptical shape by means of the open wound of longitudinal cuts or the overflowing cell building of transversal cuts. Because the DMA software uses area as a perfect circle to calculate the mechanical parameters, an “equivalent circle diameter” was calculated for the controls and the wounded stems. Dynamic mechanical tests (TA Instruments DMA Q800 Dynamic Mechanical Analyzer) were carried out on the segments, in tensile configuration and strain sweep mode, at 25–28°C, with an excitation frequency of 1 Hz and a pre-load force of 0.01 N. The two ends of each segment were tightened in the fixed and the moveable clamp, using a screwdriver Snap-on USA QDriver3 to ensure a constant fixation torque of 34 N cm for all specimens. The fixation torque was chosen so that the segments were held firmly in the clamps, but not excessively squeezed at the ends. The initial length of the specimens (length between the clamps) was 13–16 mm, and the amplitude of deformation was increased from 1 to 17 μm, corresponding to strain of 0.006% to 0.13%. The strain was calculated as the ratio between the amplitude of deformation and the initial length of the specimen. In this dynamic oscillation test method the raw signals of force, amplitude of deformation and phase angle were measured. These parameters were used to calculate the storage stiffness (K'), which corresponds to the in-phase force response of the material to the imposed sinusoidal deformation, and loss stiffness (K''), which corresponds to the out-of-phase force response of the material. Tan Delta is then calculated as the ratio of K'' to K'. Finally, the following relationship between the stiffness in tension and the tensile elastic modulus is used:

$$K=\frac{AE}{L}$$ (1)

where K is the stiffness in tension (N m^-1^), E the tensile modulus (MPa), A the sample cross-sectional area (m^2^), L sample length (m). This analysis provides an average tensile modulus of the stem, considering that it is a hierarchically organised composite material composed of several phases (tissues, cells, fibres, cell walls), which scale is below the overall scale of the samples.

The tensile storage and loss moduli are obtained as follows:

$$E\prime =K_{s}\prime \times \frac{L}{A}$$ (2)

and

$$E\prime \prime =K_{s}\prime \prime \times \frac{L}{A}$$ (3)

where E', E'' are respectively the Storage and Loss Modulus (MPa), and K_s_', K_s_'' the measured Storage and Loss Stiffness of the sample (N m^-1^).

The cross-sectional area A, to be introduced in Eqs 2 and 3 to obtain the Storage and Loss Moduli (MPa) was calculated using the diameters of the segments measured as described above. We analysed in this study both Stiffness, which is a direct measurement from the DMA force sensors, and the Moduli, which are all calculated including the variability of the diameters, but which are often considered in mechanical analysis.

The number of replications for the cv. Drakkar were n = 18 for the control stems, n = 16 for the stems with a longitudinal wound and n = 16 for the stems with a transversal wound. The number of replications for the cv. Eden were n = 18 for the control stems, n = 18 for the stems with a longitudinal wound and n = 18 for the stems with a transversal wound (raw data see S1 Table).

### Anatomical studies

Each tested segment was fixed in a Glutaraldehyde solution and embedded in resin. Cross-sections of segments were prepared with diamond-blade microtome at thicknesses of 3 μm and 1 μm and mounted on microscope slides. Thin sections of 1 μm were stained with Acridine Orange highlighting lignified tissues in yellow to green. Sections of 3 μm were stained with an overview-staining comprising a mix of Safranin, Acide Yellow, and Methylene Blue showing lignified tissues in red. Microscopic pictures were performed with an Olympus BX 61. A Zeiss filter set FITC (Excitation 495 nm, Emission 517 nm) was used for the fluorescent staining. Cross sections of approximatively 0.3 mm from fresh material were sectioned by hand with a razor blade. In order to double check lignified and non-lignified tissues, two staining were used: Fuchsin-Chrysoidin-Astrablue (FCA) highlighting lignified tissues in red-pink; and hydrochloric acid (10%) and phloroglucinol (3%) in 95% ethanol highlighting specifically lignified tissues in red. FCA pictures were performed with an Olympus BX 61 and phloroglucinol pictures with a D50 Nikkon camera and AF-S Micro Nikkor 60 mm lens.

### Statistical analysis

Mechanical properties (i.e. Storage Modulus, Stiffness and Tan Delta) were analysed in relation to strain amplitude (0.005 to 0.12%), wound type (Control, Longitudinal and Transversal) and *Linum usitatissimum* cultivar (cv. Eden and Drakkar). Morphological traits (i.e. plant height and diameter) were only investigated in relation to wound type and *Linum usitatissimum* cultivar (see S1 Fig in Supporting Information). Because we measured repeated mechanical properties (through strain sweep mode) for each stem, we carried out the analysis using linear mixed-effects models using the function *lmer* (lme4 package) in the statistical software R [29] following the model-building methods for repeated measures and longitudinal data [30, 31]. Linear mixed-effects models were also indicated to analyse morphological traits especially to take biological variability into account (random factors).

**Fig 3:**
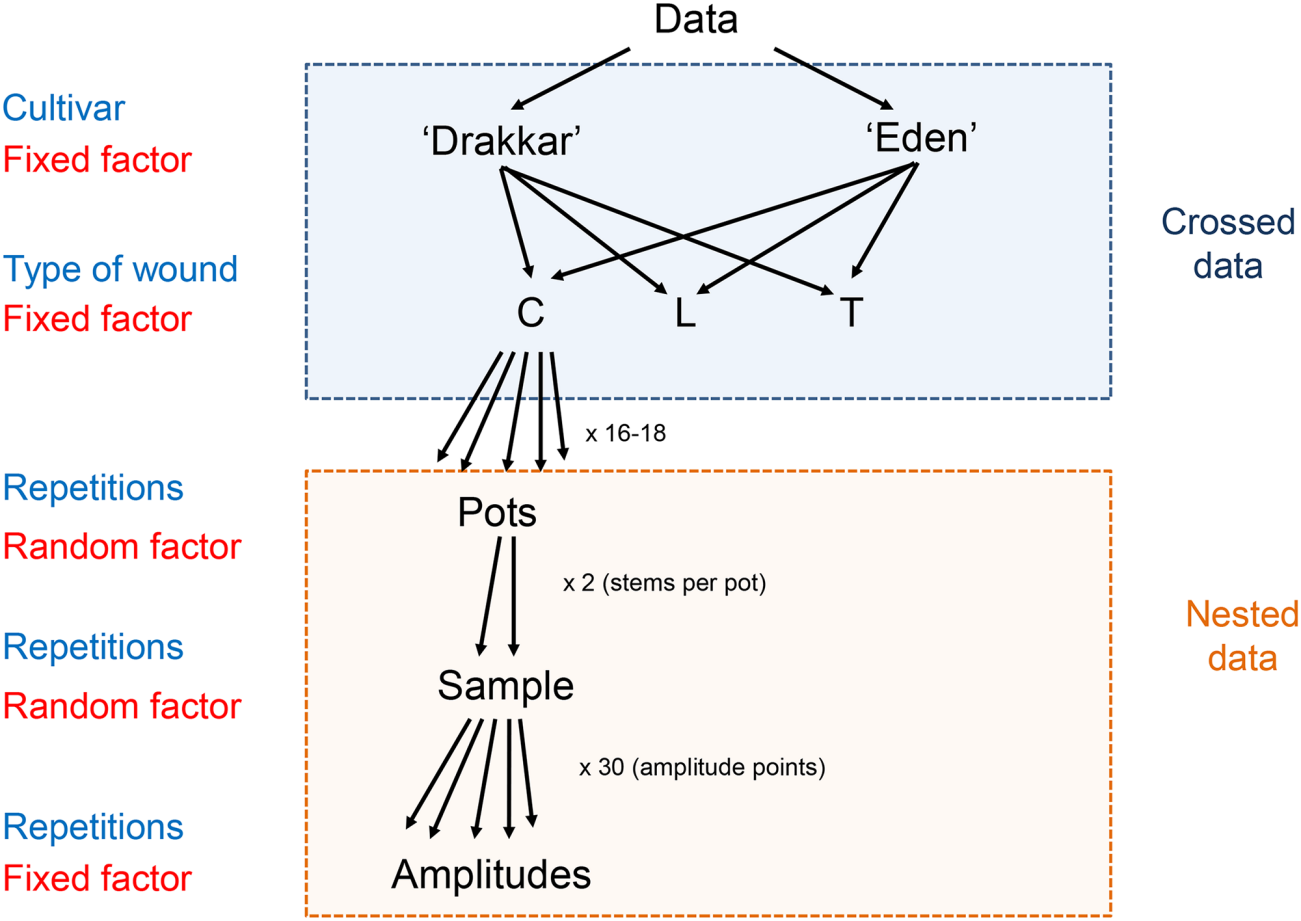
Detail of the data structure of *Linum usitatissimum* cv. Drakkar and cv. Eden showing the fixed factors and random factors with crossed and nested data.

We were mainly interested in the effect of wound type and cultivar on the mechanical properties after healing. Therefore "wound type" and "cultivar" were treated as fixed effects. The parameters "pots" and "samples" were treated as random effects where "samples" are nested into "pots" as each pot contained two plants. The parameter "Strain", calculated from the strain amplitude, was treated as a fixed effect nested into "samples" (Fig 3). We obtained full models where the fixed effect "Strain" was nested into random effects (see S1 Fig in Supporting Information). Both random slopes and random intercepts were included in the full models to take into account any variability at the level of wound type, *Linum* cultivar and Strain. In other words, we allowed the response (e.g. mechanical properties) to vary differently to each wound type, or *Linum* cultivar or Strain in order to reflect the biological variability in plants. Only significant parameters were retained for the final models (see S1 Fig in Supporting Information). The significance of both random and fixed effects was judged by performing likelihood ratio tests and comparing AIC (Akaike Information Criterion) and BIC (Schwarz/Bayesian Information Criterion) values between models. The relevance of random parameters was also tested by comparing models without random effects (linear model) and with random effects (linear mixed-effects model) with likelihood ratio tests performed with parametric bootstrap approach [31]. When necessary, optimizer options were used to solve convergence problems with the optmix package. Furthermore, the function *mstep* (lmerTest package) was used to perform a final check of the significant parameters to be retained. All estimates were taken from the final model in each case and used to draw final models on the raw data. Finally, the function *lsmeans* was used to assess the significant difference between "wound type" and "cultivar" (Post Hoc tests) and to obtain the true means (least square means) with their 95% confidence intervals. Least squares means were calculated from the final models, therefore took into account all factors (both fixed and random effects). The variables Storage Modulus, Stiffness versus Strain were all log-transformed to meet the expected relationship on log-log scale. However, means (least squares means) are presented on the original scale with their 95% confidence interval (CI). The relationship of Tan Delta versus Strain was not log-transformed as the transformation did not improve significantly the fitting. Plant Height and Diameter were also not log-transformed.

## Results

### Stem morphology and anatomy

**Fig 4:**
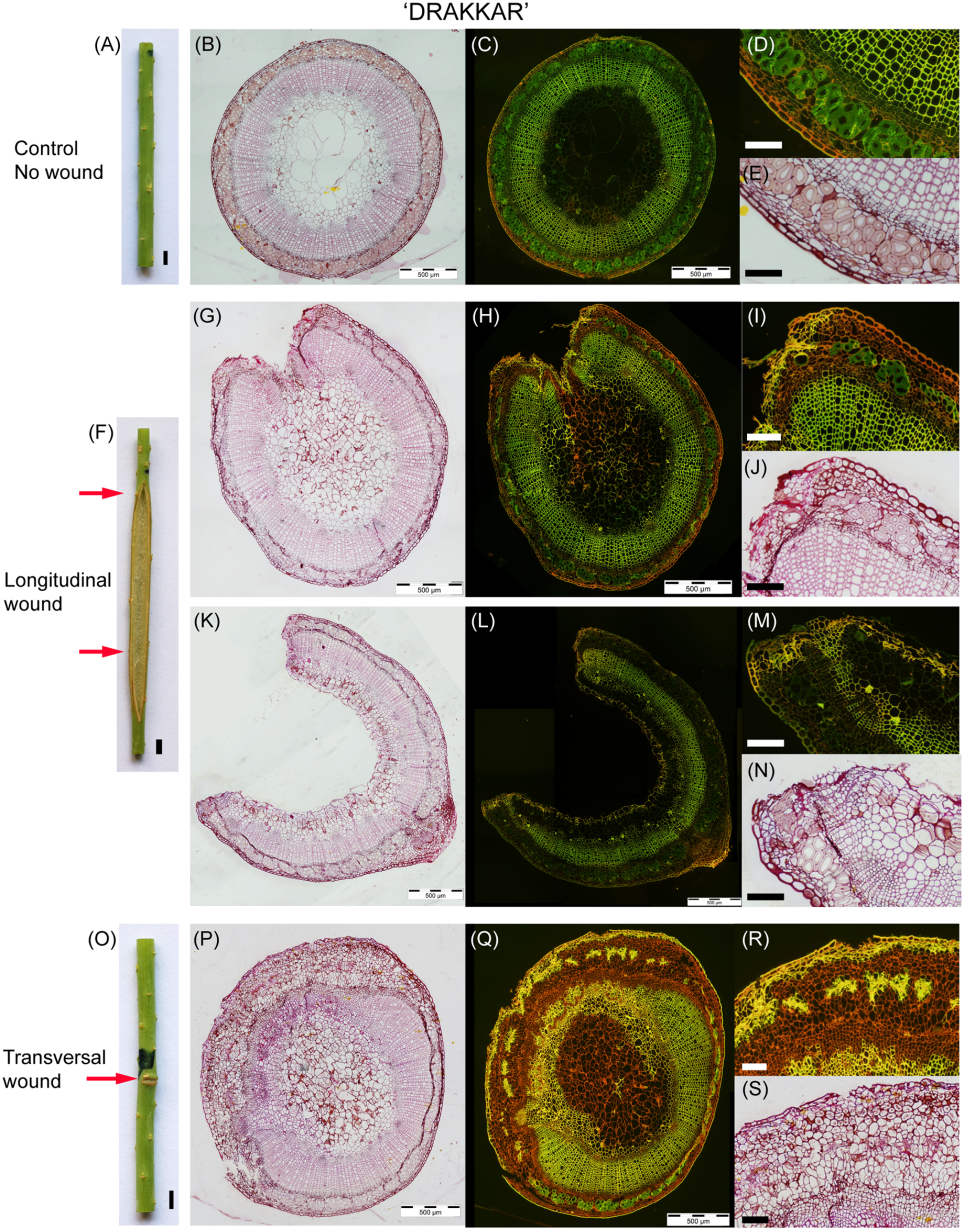
Wound morphology and stem cross sections panel of *Linum usitatissimum* cv. Drakkar. Stems at 95 DAS (A-E) control stem without wound, (F-N) 26-day-old wound parallel to the fibres and (O-S) 26-day-old wound transversal to the fibres. Staining: (B,E,G,J,K,N,P,S) Safranin, Acid Yellow and Methyleneblue. (C,D,H,I;L,M,Q,R) Acridine Orange. Scale bars: (A,F,O) = 2 mm, (D,E,I,J,M,N,R,S) = 100 μm.

**Fig 5:**
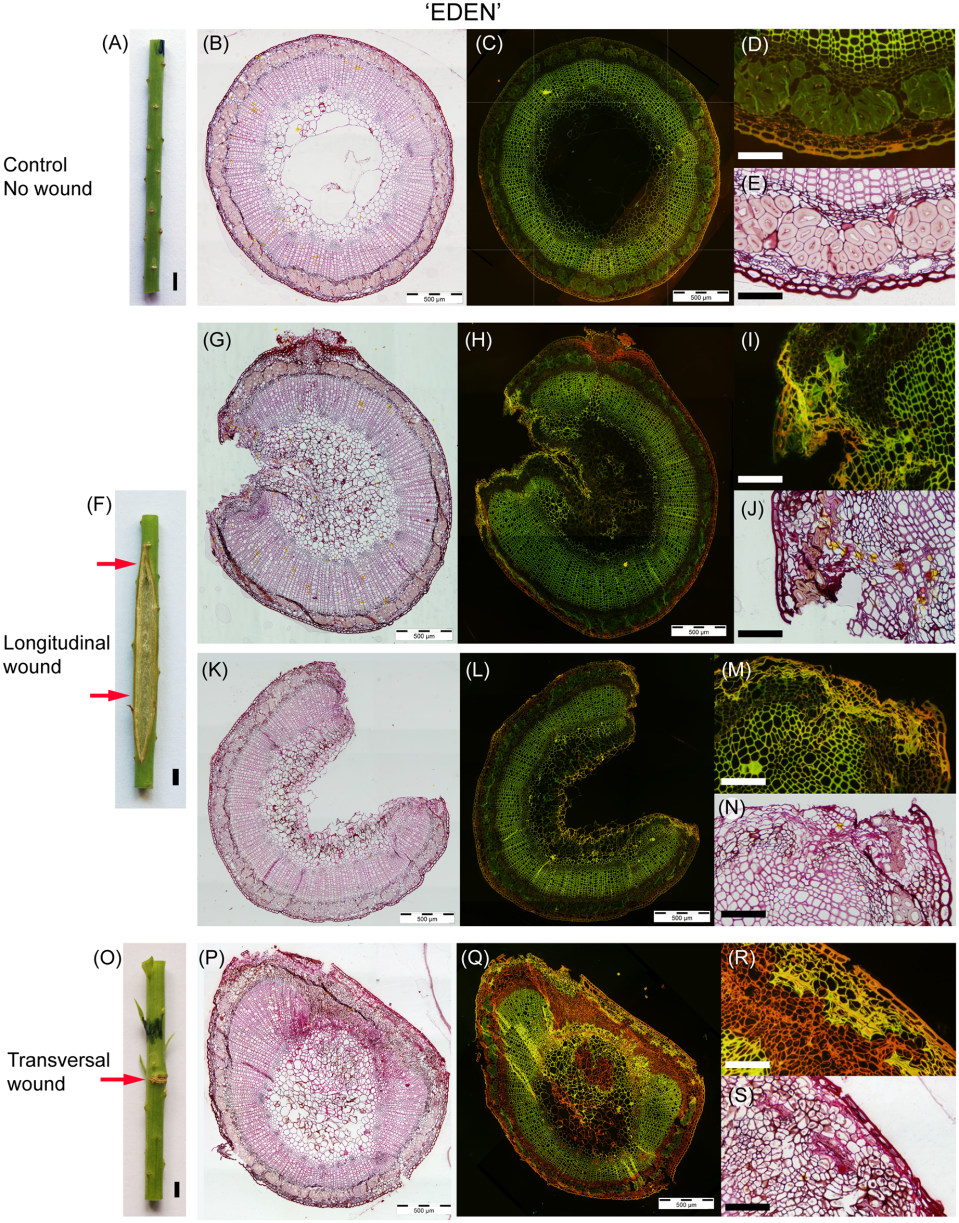
Wound morphology and stem cross sections panel of *Linum usitatissimum* cv. Eden. Stems at 95 DAS (A-CE) control stem without wound, (FD-HN) 26-day-old wound parallel to the fibres and (OI-KS) 26-day-old wound transversal to the fibres. Staining: (B,E,G,J,K,N,P,S) Safranin, Acid Yellow and Methyleneblue. (C,DF,H,I;KL,M,Q,R) Acridine Orange. Scale bars: (A,DF,O) = 2 mm, (D,E,I,J,M,N,R,S) = 100 μm.

Wound morphology and stem anatomy of both *Linum usitatissimum* cv. Drakkar and Eden are presented in Figs 4 and 5, respectively. This selection was chosen as representative from all samples. The two mechanical injuries resulted in two distinct wound healing responses for both *Linum usitatissimum* cv. Drakkar and Eden. Longitudinal incisions formed open wounds (Drakkar: Fig 4D–4H, Eden: Fig 5D–5H) while transversal incisions generated stem growth closing the wound (Drakkar: Fig 4I–4K, Eden: Fig 5I–5K). Injuries did not prevent stem development during wound healing although it reduced its final length as will be demonstrated in a later section (see "*Plant height at 95 DAS"*).

The injured stems also differed considerably from the controls in terms of tissue organisation and histology. *Linum usitatissimum* control stems consist of an epidermis, a cortex, bundles of bast fibres (sclerenchyma fibres), phloem, cambium, xylem and pith (Drakkar: Fig 4B and 4C, Eden: Fig 5B and 5C) forming continuous rings except for the pith. *Linum* bast fibres have a low lignin content of 5% maximum [32] resulting in mainly non-lignified sclerenchyma bundles distributed in the stem periphery. Fluorescent staining supported the minor lignin content of sclerenchyma cells in both cultivars (Drakkar: Fig 4C, Eden: Fig 5C) compared to the highly lignified xylem.

**Fig 6:**
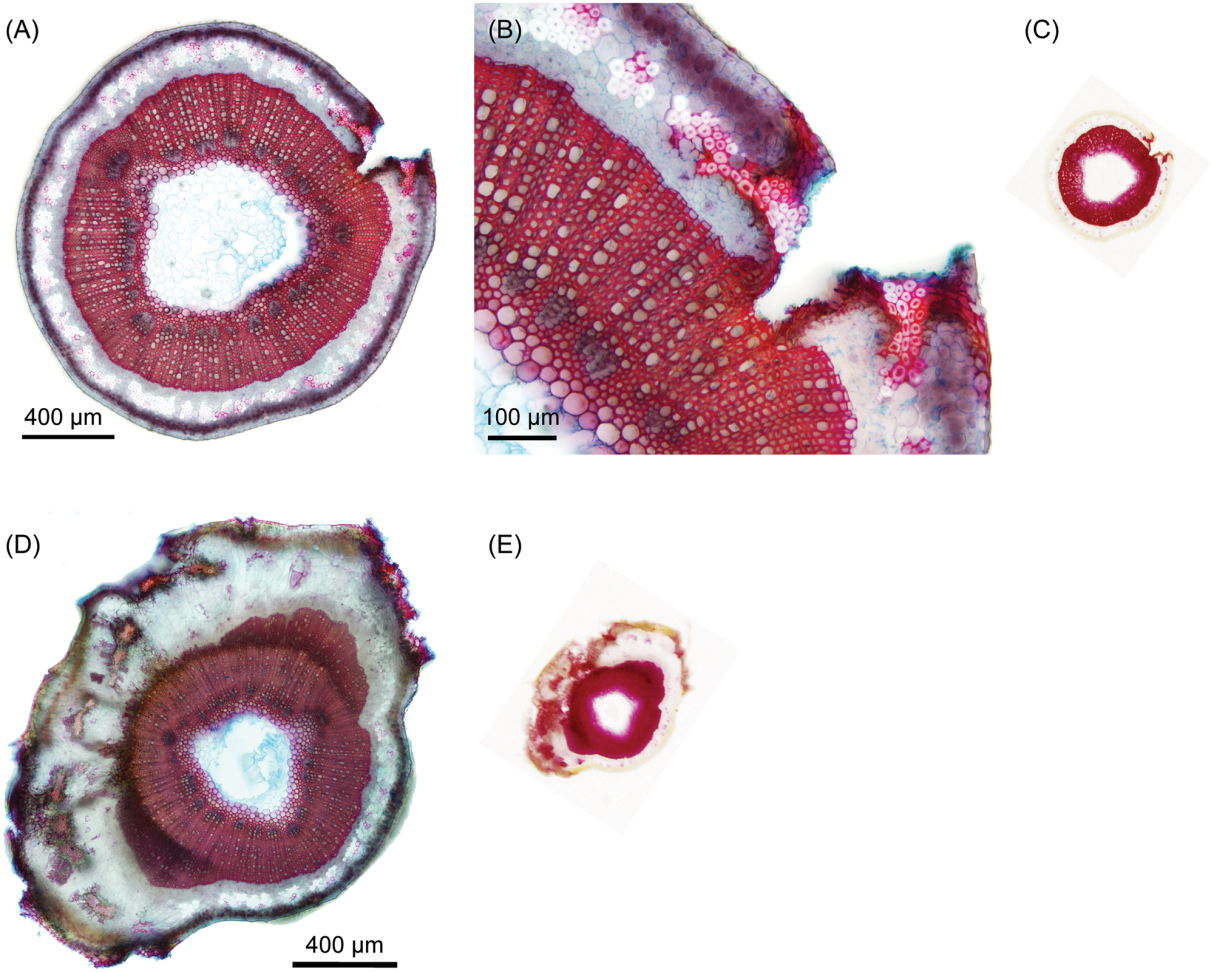
Stem cross sections of *Linum usitatissimum* showing wound tissue lignification. (A-C) Stem cross section of a transversal wounded stem and (D-E) stem cross section of a longitudinal wounded stem. Staining: (A,B,D) Fuchsin-Chrysoidin-Astrablue, (C,E) Phloroglucinol.

The anatomy of the longitudinal wounds locally affected the stem around the incision (Drakkar: Fig 4G–4J, Eden: Fig 5G–5J) showing structural changes in cells bordering the injured tissues, the rest of the cross section remaining comparable to the control plants. The tissues injured with the incision (i.e. cortex, bast fibres, phloem and part of the xylem) were not restored. Instead, a protective boundary layer is observed along the opened wound (suberin and lignin deposition). Below, a layer of parenchymatous cells (i.e. wound periderm) has developed. Some of the bast fibre bundles bordering the wound became also lignified (Figs 4I, 4J and 6A–6C).

In the middle of the wound, where wound edges are completely apart, main changes are the severe loss of a major part of the cross section and the development of a protective barrier (suberin and lignin deposition) on the wound edges and inside along the pith (Drakkar: Fig 4K–4N, Eden: Fig 5K–5N). Tissue organisation remains remarkably the same in the remaining stem tissues. Again, some of the bast fibre bundles bordering the wound are also lignified (Fig 5I and 5J).

In the case of transversal wound healing, tissue organisation is profoundly perturbed with a larger region of the stem being affected (Drakkar: Fig 4P–4S, Eden: Fig 5P–5S). The injured tissues (i.e. cortex, bast fibres, phloem and part of the xylem) show a complete closing of the wound thus restoring the whole cross section but not the original stem organisation. Wound healing stimulated a ligno-suberized deposition in the epidermis and part of the cortex forming a protective barrier. All the bast fibre bundles in the perturbed part remained cut up without reconnection of the fibre ends but became lignified and pulled apart by parenchyma cells growth while the undamaged bundles remained non-lignified with a regular distribution in the periphery. The phloem and cambial tissues are greatly disturbed and a pronounced formation of parenchyma cells and narrows vessels occurs. Some non-lignified narrow vessels are observed within the xylem could possibly be remains of former cambium before incision. Vascular vessels of the phloem and xylem present an organisation parallel to the incision instead of a classic radial orientation for the xylem. Pith is also affected with some cells showing lignification (Fig 6D and 6E).

### Calculating the model parameters and fitting models

#### Storage modulus

**Fig 7:**
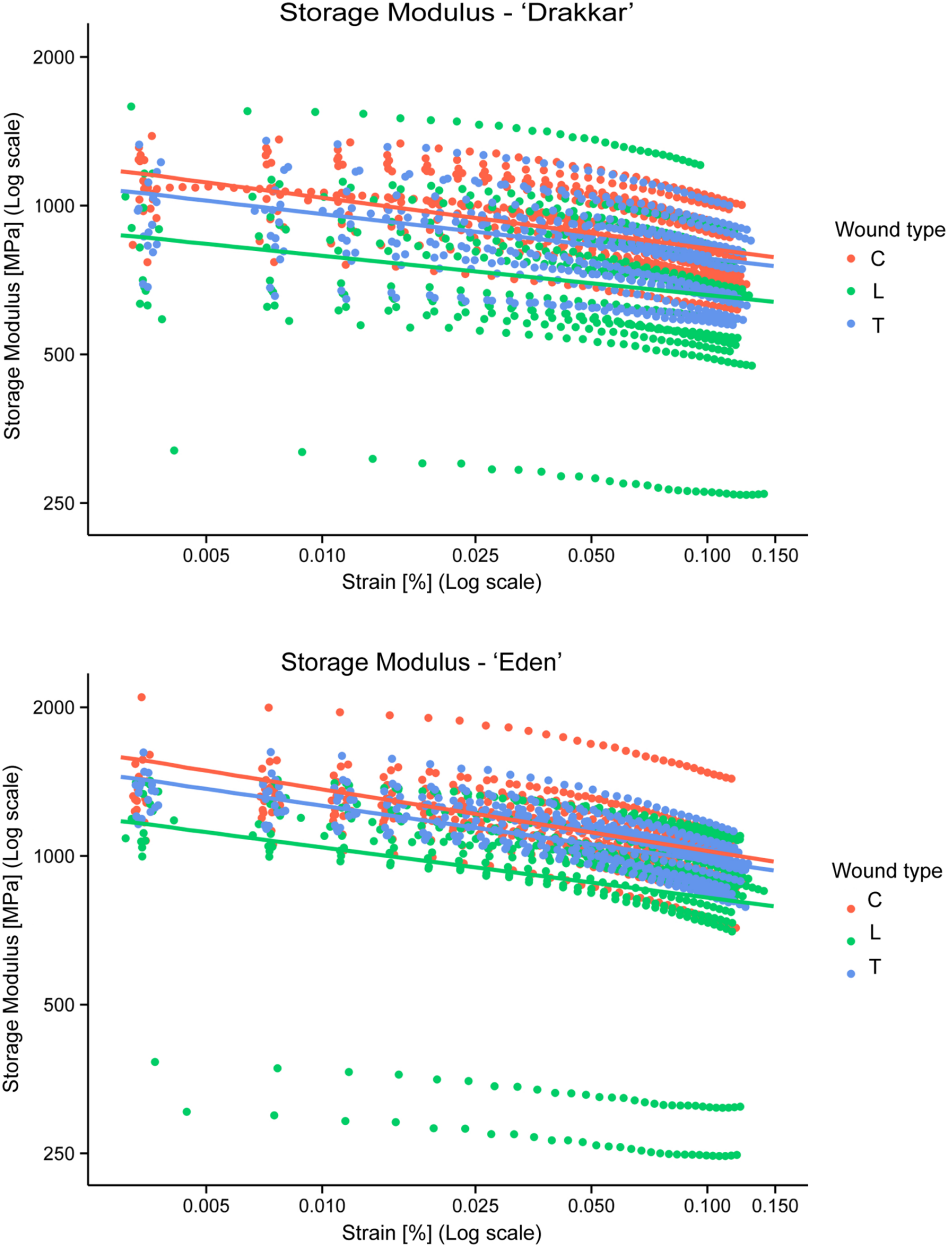
Measured storage modulus (plain colour circles) versus strain and the final model from the lmer model with fixed effects (solid colour lines) for both *Linum usitatissimum* cv. Drakkar and cv. Eden. All parameters were log-transformed. Legend: type is type of wound (C: Control/no wound, L: Longitudinal wound, T: Transversal wound).

The relationship between Storage Modulus and Strain was significantly negative for both cultivars and all wound types (Fig 7; Table 1a). Strain significantly affected the Storage Modulus (χ^2^(4) = 386.19, *p* < 0.0001) indicating that Storage Modulus decreased with Strain in all cases.

**Table 1:** Estimates of the fixed effects from the final linear mixed effects models fitted against type of wound, cultivar and amplitude. Non-significant terms were removed. Random i.e. sample effects were retained (see S1 Fig in Supporting Information). The approximation of degrees of freedom is Satterthwaite's. LogSTRAIN is Log(Strain), type is type of wound (L: Longitudinal wound, T: Transversal wound), cv is cultivar of *Linum usitatissimum* (ED: cv. Eden), SE is standard error.

	Estimate	SE	df	*t*-value	*P*-value
**(a) Storage Modulus (MPa)**					
Intercept	6.98	0.04538	104	142.62	< 0.0001
LogSTRAIN	-0.102	0.00316	104	-32.46	< 0.0001
type L	-0.164	0.05612	104	-2.93	< 0.01
type T	-0.0169	0.05612	104	-0.30	0.76
cvED	0.172	0.04606	104	3.73	< 0.0001
LogSTRAIN: typeL	0.0230	0.00391	104	5.90	< 0.0001
LogSTRAIN: typeT	0.0128	0.00391	104	3.29	< 0.01
LogSTRAIN: cvED	-0.0221	0.00321	104	-6.90	< 0.0001
**(b) Stiffness (N/m)**					
Intercept	11.81	0.06069	104	194.59	< 0.0001
LogSTRAIN	-0.102	0.00317	104	-32.35	< 0.0001
type L	-0.245	0.07505	104	-3.26	< 0.01
type T	-0.117	0.07505	104	-1.5551	0.12
cvED	0.282	0.06160	104	4.59	< 0.0001
LogSTRAIN: typeL	0.0230	0.00392	104	5.87	< 0.0001
LogSTRAIN: typeT	0.0128	0.00392	104	3.26	< 0.01
LogSTRAIN: cvED	-0.0221	0.00322	104	-6.89	< 0.0001
**(c) Tan Delta (/)**					
Intercept	0.108	0.00203	104	53.35	< 0.0001
strain	0.844	0.02648	104	31.88	< 0.0001
type L	-0.00597	0.00251	104	-2.38	< 0.05
type T	-0.00859	0.00251	104	-3.42	< 0.001
cvED	0.000203	0.00206	102	0.10	0.922
strain: typeL	-0.0932	0.03274	104	-2.85	< 0.01
strain: typeT	-0.0228	0.03273	104	-0.70	0.487
strain: cvED	0.0974	0.02686	104	3.62	< 0.001
**(d) Plant height (cm)**					
Intercept	74.97	0.690	377	108.72	< 0.0001
type L	-9.492	0.994	853	-9.55	< 0.0001
type T	-3.379	0.967	734	-3.49	< 0.001
cvED	20.33	0.979	1731	20.77	< 0.0001
TypeL: cvED	-8.118	1.397	2222	-5.81	< 0.0001
TypeT: cvED	-4.453	1.378	2114	-3.23	< 0.01
**(e) Diameter (mm)**					
Intercept	1.938	0.0174	109	111.56	< 0.0001
type L	0.03846	0.0248	191	1.55	0.1226
type T	-0.06198	0.0239	171	-2.60	0.0103
cvED	0.2159	0.0246	206	8.77	< 0.0001
TypeL: cvED	-0.2574	0.0350	248	-7.36	< 0.0001
TypeT: cvED	-0.1025	0.0343	242	-2.99	< 0.01

The cultivar of *Linum* significantly affected the Storage Modulus (χ^2^(2) = 44.47, *p* < 0.0001). Eden cultivar produced stems with greater Storage Modulus compared to Drakkar cultivar (*t*(108.16) = -4.86, *p* < 0.0001 from Tukey Post hoc tests). For Drakkar stems E' was lower by around 21.3% compared to Eden stems (Tables 2a and 3).

**Table 2:** Average (Least squares means) mechanical properties and morphological traits for each wound type presented with 95% confidence interval for both *Linum usitatissimum* cultivars. Calculated from the final linear mixed effect model (see S1 Fig in Supporting Information) taking into account both fixed and random effects. The mechanical properties were obtained with a Strain sweep from 0.003% up to 0.14%.

Properties/Traits	Wound type	*n*	cv. Eden	*n*	cv. Drakkar
(a) Storage modulus (MPa)	Control	18	1127.00 [1023.47–1241.01]	18	886.74 [805.28–976.44]
	Longitudinal	18	890.84 [808.44–981.62]	16	700.92 [634.28–774.56]
	Transversal	18	1065.23 [966.71–1173.79]	16	838.13 [758.44–926.19]
(b) Stiffness (N/m)	Control	18	262 602 [228 808–299 096]	18	184 239 [161 143–210 645]
	Longitudinal	18	190 863 [166 778–218 428]	16	134 419 [116 992–154 445]
	Transversal	18	223 816 [195 571–256 138]	16	157 628 [137 190–181 108]
(c) Tan Delta (/)	Control	18	0.1653 [0.1612–0.1694]	18	0.1592 [0.1551–0.1634]
	Longitudinal	18	0.1537 [0.1496–0.1579]	16	0.1477 [0.1434–0.1519]
	Transversal	18	0.1553 [0.1512–0.1595]	16	0.1493 [0.1450–0.1535]
(d) Plant Height (cm)	Control	18	95.3 [93.9–96.7]	18	75.0 [73.6–76.3]
	Longitudinal	18	77.7 [76.3–79.1]	16	65.5 [64.1–66.9]
	Transversal	18	87.5 [86.1–88.8]	16	71.6 [70.3–72.9]
(e) Stem diameter (mm)	Control	18	2.15 [2.12–2.19]	18	1.94 [1.90–1.97]
	Longitudinal	18	1.94 [1.90–1.97]	16	1.98 [1.94–2.01]
	Transversal	18	1.99 [1.95–2.02]	16	1.88 [1.84–1.91]

**Table 3:** Self-healing efficiency according to wound type and *Linum usitatissimum* cultivar calculated from the least squares means of the final models. This summary table is possible because the fixed effects wound type and *Linum* cultivars are non-interdependent for all mechanical properties.

		Storage modulus (MPa)	Stiffness (N/m)	Tan Delta (/)
Wound type	Control	100%	100%	100%
	Longitudinal	79.04%	72.96%	92.86%
	Transversal	94.52%	85.56%	93.86%
*Linum* cultivar	Eden	100%	100%	100%
	Drakkar	78.68%	70.43%	96.16%

The type of wound also significantly affected the Storage Modulus (χ^2^(4) = 35.50, *p* < 0.0001). The average Storage Modulus decreased with both longitudinal and transversal wound compared to control stems without wound (Table 2a). Transversal wound healing recovered the Storage Modulus most efficiently, closely approaching the control properties (*t*(108.16) = 0.94, *p* = 0.62 from Tukey Post hoc tests), whereas in stems with longitudinal wound recovery of Storage Modulus was significantly lower than controls (*t*(108.16) = 3.91, *p* = 0.0005) and significantly lower than in the stems with transversal wounds (*t*(108.16) = -2.93, *p* = 0.011). Stems with longitudinal and transversal wounds showed lower mechanical properties by around 21.0% and 5.5% respectively compared to control stems (Table 3). Thus wound-healing could not recover completely the Storage Modulus.

Interestingly, the *Linum* cultivar × wound type interaction was not significant (χ^2^(2) = 0.50, *p* = 0.78). It indicated that *Linum* cultivar and wound type were not inter-dependent and although both cultivars showed different properties, they both reacted the same way to the different wound types. This interaction was therefore removed from the final model. In contrast *Linum* cultivar × Strain (χ^2^(1) = 39.13, *p* < 0.0001) and wound type × Strain (χ^2^(2) = 30.21, *p* < 0.0001) interactions were significant and thus kept in the model. Furthermore, the *Linum* cultivar × wound × Strain interaction was not significant (χ^2^(2) = 3.31, *p* = 0.192) and therefore removed from the model. For the random effects, the pot effect was not significant (χ^2^(78) = 22.70, *p* = 1) and was therefore removed from the model. Also, having two stems per pot did not affect their properties. In contrast, the random effect of sample was highly significant (from bootstrapping approach, *p* < 0.0001) indicating the marked biological variability between *Linum* stems. Keeping random effect was essential to the model. The table of estimates from the final lmer model from Storage Modulus is shown in Table 1a and the data plotted together with the model fitting in Fig 7.

#### Stiffness

**Fig 8:**
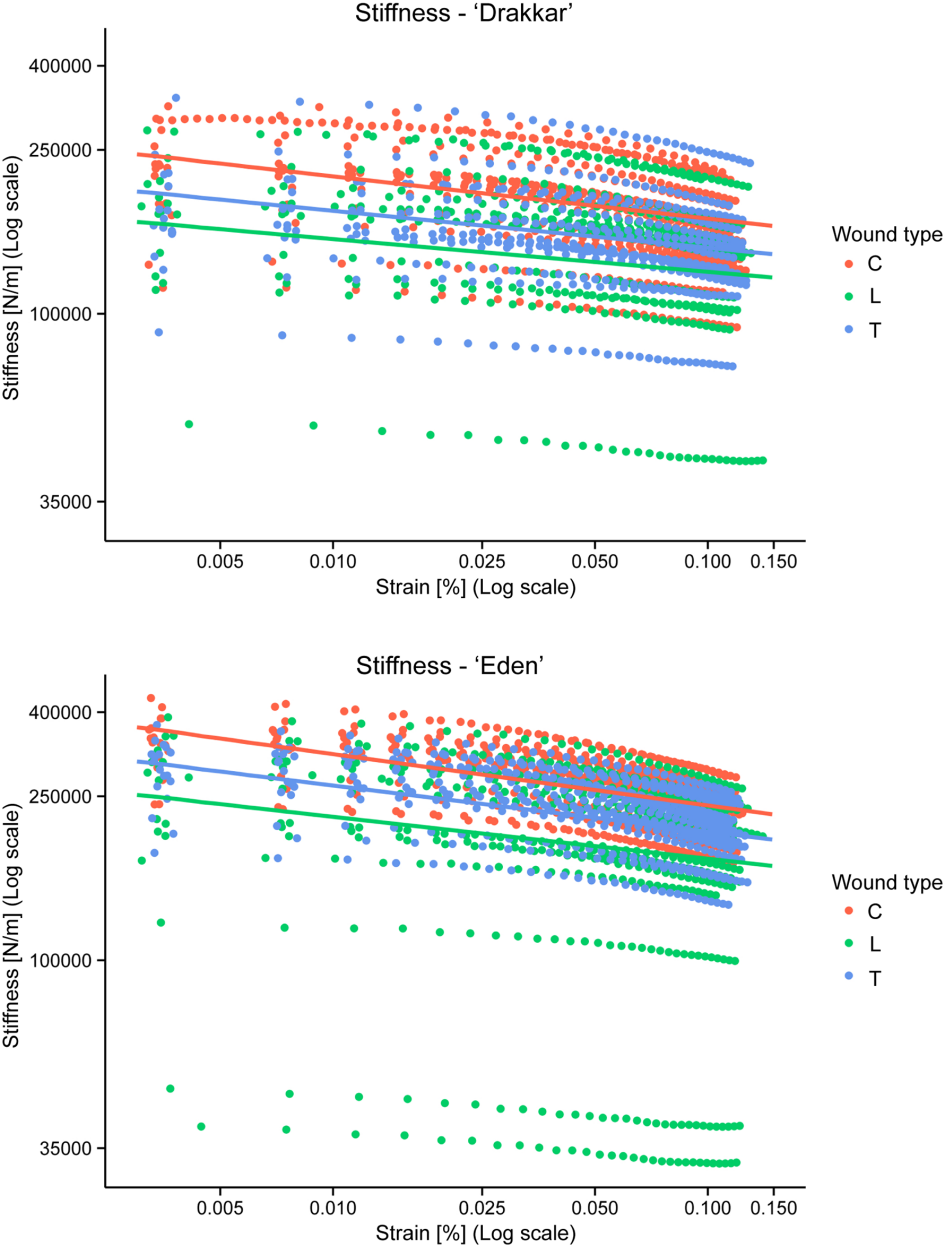
Measured stiffness (plain colour circles) versus strain and the final model from the lmer model with fixed effects (solid colour lines) for both *Linum usitatissimum* cv. Drakkar and cv. Eden. All parameters were log-transformed. Legend: type is type of wound (C: Control/no wound, L: Longitudinal wound, T: Transversal wound).

The relationship between Stiffness and Strain was significantly negative for both cultivars and all wound types (Fig 8; Table 1b). Strain significantly affected Stiffness (χ^2^(4) = 385.58, *p* < 0.0001) indicating that Stiffness was decreasing with Strain in all cases.

The cultivar of *Linum* significantly affected Stiffness (χ^2^(2) = 39.99, *p* < 0.0001). Eden cultivar produced stems with higher Stiffness compared to Drakkar (*t*(108.16) = -5.111, *p* < 0.0001 from Tukey Post hoc tests). On average for Drakkar stems this mechanical property was lower by around 29.6% compared to Eden stems (Tables 2b and 3). The type of wound also significantly affected Stiffness (χ^2^(4) = 30.12, *p* < 0.0001). The average Stiffness decreased with both longitudinal wound and transversal wound compared to control stem without wound (Table 2b). Transversal wound healing recovered Stiffness the most efficiently approaching the control properties being not significantly lower (*t*(108.16) = 1.87, *p* = 0.153 from Tukey Post hoc tests), whereas Stiffness of stems with longitudinal wound recovery was significantly lower than controls (*t*(108.16) = 3.77, *p* = 0.0008). In contrast to Storage Modulus, Stiffness of stems with longitudinal wound was not significantly lower than those with transversal wound (*t*(108.16) = -1.88, *p* = 0.150). Longitudinal wounds and transversal wounds lead to a lower stiffness by around 27.0% and 14.4%, respectively, compared to control stems (Table 3). Thus wound-healing could not completely recover the stem Stiffness.

Interestingly, as seen for the Storage Modulus, the *Linum* cultivar × wound type interaction was not significant (χ^2^(2) = 0.86, *p* = 0.65). This interaction was therefore removed from the final model. In contrast *Linum* cultivar × Strain (χ^2^(1) = 39.01, *p* < 0.0001) and wound type × Strain (χ^2^(2) = 29.97, *p* < 0.0001) interactions were significant and thus kept in the model. Furthermore, the *Linum* cultivar × wound × Strain interaction was not significant (χ^2^(2) = 3.29, *p* = 0.193) and therefore removed from the model. As for Storage Modulus, the pot effect was not significant (χ^2^(78) = 15.46, *p* = 1) and the random effect of sample was highly significant (from bootstrapping approach, *p* < 0.0001). The table of estimates from the final lmer model from Stiffness is shown in Table 1b and the data plotted together with the model fitting in Fig 8.

#### Tan Delta

**Fig 9:**
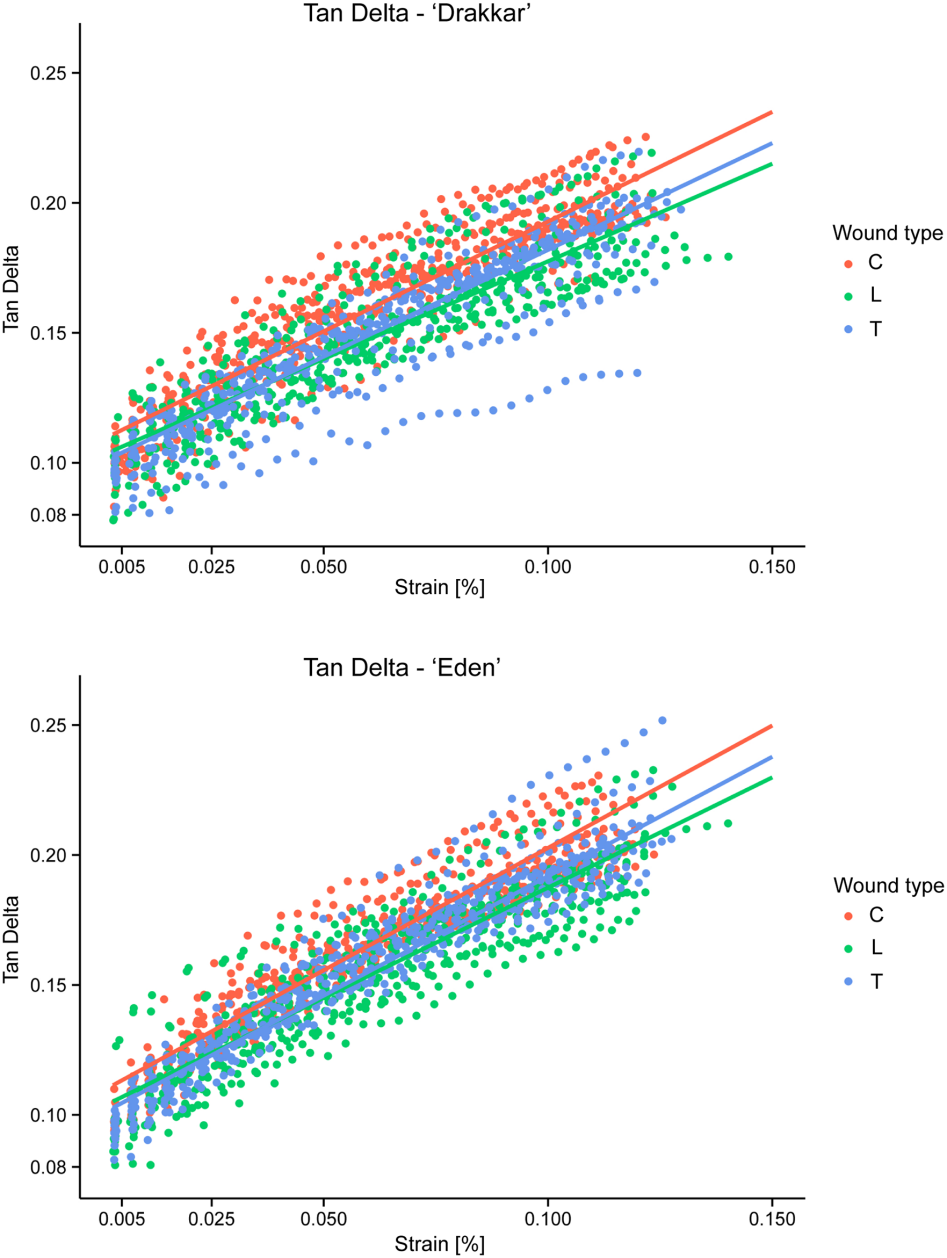
Measured Tan Delta (plain colour circles) versus strain and the final model from the lmer model with fixed effects (solid colour lines) for both *Linum usitatissimum* cv. Drakkar and cv. Eden. All parameters were log-transformed. Legend: type is type of wound (C: Control/no wound, L: Longitudinal wound, T: Transversal wound).

The relationship between Tan Delta and Strain was significantly positive for both cultivars and all wound types (Fig 9, Table 1c). Strain significantly affected Tan Delta (χ2(4) = 384.79, p < 0.0001) indicating that Tan Delta increased with greater Strain in all cases.

Cultivar of *Linum* significantly affected the Tan Delta (χ2(4) = 21.86, *p* < 0.001). Eden cultivar produced stems with higher Tan Delta compared to Drakkar (t(108.16) = -5.11, p < 0.0001 from Tukey Post hoc tests). For Drakkar stems this mechanical property was lower by around 3.8% compared to Eden stems (Tables 2c and 3). Type of wound significantly affected the Tan Delta (χ^2^(4 = 26.51, *p* < 0.0001). The average Tan Delta of stems with longitudinal and transversal wounds decreased compared to control stems without wound (Table 2c). Transversal wound healing recovered slightly better Tan Delta but was significantly lower (*t*(108.16) = 3.77, *p* < 0.001 from Tukey Post hoc tests). Longitudinal wound recovery was not significantly lower than controls (*t*(108.16) = 1.87, *p* = 0.15). In contrast to Storage Modulus, Tan Delta of stems with longitudinal wound was not significantly lower than those with transversal wound (*t*(108.16) = -1.88, *p* = 0.15). Stems with longitudinal and transversal wounds showed Tan Delta values lower by around 7.1% and 6.1% respectively compared to control stems (Table 3). Thus wound-healing could not recover completely Tan Delta. As for both Storage Modulus and Stiffness, the *Linum* cultivar × wound type interaction was not significant (χ^2^(2) = 1.59, *p* = 0.45), and this interaction was therefore removed from the final model. In contrast *Linum* cultivar × Strain (χ^2^(1) = 12.36, *p* < 0.001) and wound type × Strain (χ^2^(2) = 0.15, *p* = 0.015) were significant and thus kept in the model. Furthermore, the *Linum* cultivar × wound × Strain interaction was not significant (χ^2^(2) = 1.20, *p* = 0.55) and therefore removed from the model. For the random effects, the pot effect was not significant (χ^2^(78) = 20.15, *p* = 0.99) and the random effect of sample was highly significant (from bootstrapping approach, *p* < 0.0001) indicating the biological variability between *Linum* stems. Keeping random effect was essential to the model. The table of estimates from the final lmer model from Tan Delta is shown in Table 1c and the data plotted together with the model fitting in Fig 9.

#### Plant height at 95 DAS

The relationship between plant height at 95 DAS and type of wound was significantly negative (Table 1d) for both stems with longitudinal and transversal wounds indicating that plant height decreased when damaged with wounds. The *Linum* cultivar × wound type interaction was highly significant (χ2(2) = 17057, p < 0.0001) indicating a strong inter-dependence of the two fixed factors in contrast to the mechanical properties. Plant height for Drakkar cultivar (Table 2d) was significantly lower with a longitudinal wound by around 12.7% (t(537.2) = 9.56, *p* < 0.0001 from Tukey Post hoc tests) and 4.5% with a transversal wound (*t*(310.4) = 3.50, *p* < 0.0001) compared to the control Drakkar plants. The two types of wounds differed significantly (*t*(305.9) = -6.20, *p* < 0.0001). Plant height for Eden cultivar (Table 2d) was also significantly lower with a longitudinal wound by around 18.5% (t(211.9) = 17.93, *p* < 0.0001 from Tukey Post hoc tests) and 8.2% with a transversal wound (*t*(232.0) = 7.97, *p* < 0.0001) compared to the control Eden plants. The two types of wounds differed significantly (*t*(222.9) = -9.96, *p* < 0.0001). Thus wounds affected plant height and healing could not recover the height at 95 DAS. For the random effects, the pot effect was significant (χ^2^(1) = 5136.9, *p* < 0.0001) and was therefore kept in the model nested into sample random effect. The random effect of sample was also significant (from bootstrapping approach, *p* < 0.0001) indicating the biological variability between *Linum* stems. Keeping both random effects was essential to the model. The table of estimates from the final lmer model from plant height is shown in Table 1d.

#### Diameter at 95 DAS

The relationship between tested segment diameter at 95 DAS and type of wound was mainly indicating a negative relationship apart from longitudinal wounding were the output was less significant and not negative (Table 1e). It suggested that the diameter response to wounding was not homogenous. The *Linum* cultivar × wound type interaction was highly significant (χ2(2) = 2334.8, *p* < 0.0001) indicating a strong inter-dependence of the two fixed factors in contrast to the mechanical properties. Diameter for Drakkar cultivar (Table 2e) was significantly lower by around 3.1% with a transversal wound (t(209.8) = 2.59, *p* = 0.01 from Tukey Post hoc tests) contrary to diameter with a longitudinal wound showing no significant difference (*t*(231.1) = -1.55, *p* = 0.12) compared to the control Drakkar plants. The two types of wounds differed significantly (*t*(212.4) = 4.16, *p* < 0.0001). Diameter for Eden cultivar (Table 2e) was significantly lower both with a longitudinal wound by around 9.8% (t(231.1) = -1.55, *p* < 0.0001 from Tukey Post hoc tests) and 7.4% by a transversal wound (*t*(209.8) = 2.59, *p* < 0.0001) compared to the control Eden plants. The two types of wounds differed significantly (*t*(147.3) = -2.20, *p* = 0.03). Thus tested segment diameters were mostly affected by wounds and in most of the cases, healing could not recover diameters at 95 DAS. For the random effects, the pot effect was significant (χ^2^(1) = 7652.5, *p* < 0.0001) and was therefore kept in the model nested into sample random effect. The random effect of sample was also significant (from bootstrapping approach, *p* < 0.0001) indicating the biological variability between *Linum* stems. Keeping both random effects was essential to the model. The table of estimates from the final lmer model from stem diameter is shown in Table 1e.

## Discussion

The present study describes significant differences in the mechanical, morphological and anatomical responses to mechanical damage of two *Linum usitatissimum* cultivars, one with stems resistant to lodging (cv. Eden) and one with more flexible stems (cv. Drakkar). Although mechanical properties could not be fully recovered after a 25-day healing period, both cultivars could show a healing efficiency from 79% up to 95% with higher scores in favour of transversal wound healing.

### Effect of wound directions

An important aspect about the healing efficiency is the wound direction with respect to the fibres. Sclerenchyma fibre bundles of *Linum usitatissimum* are distributed in the periphery of the stem and present a unidirectional arrangement along the stem [28, 33]. With this configuration, transversal wounds might cause greater alterations. They completely cut the fibres at right angles and might also cut the cambium at right angles to its plane of division [34], thus altering the vascular function if phloem and/or xylem are affected and markedly weaken the supporting functions of the fibres. The longitudinal wounds cause separation of the bundles while preserving the continuity of the fibres and consequently keeping more or less the supporting functions unaltered and injure fewer cells [34]. Therefore, we might expect the stems with longitudinal wounds to recover better. Interestingly, after the 25-day healing period, we observed that the stems with transversal wounds perform better in terms of mechanical properties recovery but also in terms of plant height.

At the morphological level, in the case of transversal wound healing, the injured tissues showed a complete closure thus restoring the whole cross section although not the original stem organisation. In the case of the longitudinal wound healing, injured tissues were not restored and showed structural changes in the bordering cells, where wound edges remain completely apart. An open wound might indeed disadvantage a stem with desiccation of internal tissues usually not exposed and a loss of mechanical support by the void created. In contrast, the transversal wounds responded by a local reinforcement of the wounded tissues by enhanced growth and marked histological changes including clearly visible lignification of fibre bundles.

The complete opening of the wound induced after a longitudinal incision might be partly attributed to an anisotropic distribution of (pre-)tension and compression present in the stem having a special arrangement of tissues with different mechanical properties. In the case of a transversal wound, this anisotropic distribution does not result in a permanent opening of the wound, because the upper part of the stem might constrain the wound to close down by applying its own mass. This contribution to bring the wound edges pressed together might facilitate the wound healing.

Furthermore, the incisions were performed during an early phase of development of *Linum*, where the plants were still building their fibres and were actively growing. The stem continued to increase in height and radius. This radial growth might have enhanced the opening of the longitudinal wounds by enhanced crack propagation and exposed internal tissues, thus maintaining a disadvantage in terms of recovery. The wound timing is therefore a critical element in the wound healing process and its analysis.

### Mechanical damage affects fibres quality by triggering tissue lignification

Both *Linum usitatissimum* cultivars developed protective chemical barriers during wound healing. In the case of a longitudinal wound (Figs 4D and 5D), a protective boundary layer is observed along the open wound (suberin and lignin deposition) with a layer of parenchyma cells below (i.e. wound periderm). Some of the bast fibre bundles bordering the wound became also markedly lignified. In the case of the transversal wound (Figs 4I and 5I), a ligno-suberized deposition in the epidermis and part of the cortex formed a protective barrier. All the bast fibre bundles in the perturbed part were clearly lignified and pulled apart by parenchyma cells growth while the other bundles remained non-lignified with a regular distribution in the periphery.

While the deposition of ligno-suberin material around the wound epidermis has been reported in a previous study on wound response in flax [35], the reaction of the sclerenchyma fibre bundles was not investigated. These fibres are especially important due to their role of mechanical support [25, 32] but are also of high interest to industrial use. Among the wound healing responses in our findings, a striking element is the lignification of the sclerenchyma fibre bundles in the damaged area and its vicinity. When not damaged, the *Linum* sclerenchyma fibre bundles have a low lignin content ranging from 2% to 5% [15, 23, 25, 33]. This lignin deposition might have two important consequences. First, the fibre quality could be affected because higher lignin content directly impacts the fibre quality by decreasing its strain to failure [32, 36, 37]. This parameter is so important that transgenic flax with lignin deficiency is investigated to improve elastic properties of bast fibres [37]. Damaged or wounded flax stems might therefore decrease textile quality. Secondly, our findings showed a singular case of lignin deposition triggered by mechanical wound during sclerenchyma differentiation. Lignin deposition during sclerenchyma differentiation occurs naturally in Alfalfa (*Medicago sativa* L.) undamaged stems [38] where the onset of lignification occurs in the secondary cell walls contrary to secondary xylem. A similar process might occur for the sclerenchyma fibre bundles in the injured region of the cultivars stems, explaining how the fibres already present during incision could be completely lignified although they are naturally very low in lignin content.

### Mechanical healing efficiency rate consistent between the two cultivars

The mechanical response of *Linum usitatissimum* cv. Drakkar and cv. Eden stems to increasing strain was comparable for all wound types and for controls. Both cultivars showed decreasing Storage Modulus and Stiffness at higher strains, while Tan Delta increased at higher strains. The slight decrease in Modulus and Stiffness could be attributed to some early damage in the cells of the fibres, due to an orientation of the microstructure in the strain axis. As a result, the dissipation that is observed is most likely resulting from viscous dissipation, but also to a small extent of damage in the stems. Since both cultivars, wounded or not showed the same trend, the dissipation is assumed to follow similar mechanisms in all cases.

The increase in dissipation of the stem material (represented by Tan Delta) combined with the decrease in elastic behaviour (represented by Storage Modulus) demonstrated that damping properties increase despite wounded stems. These mechanical traits at very small deformations (1 to 17 μm) reflect the role of water and also the crucial role of the sclerenchyma fibres in the stems by an increasing energy dissipation to respond to the increasing deformation. Even though the connection between flax stem properties and processed flax fibres is not straightforward in terms of intrinsic damping properties, it is worth noting that processed flax fibres composites are especially of interest for their intrinsic damping properties in particular in sport equipment [39]. Despite the loss of elasticity via the Storage Modulus decrease and the wounds, Drakkar and Eden stems were still able to enhance damping properties by increasing their dissipation at higher strains.

Furthermore, although the two cultivars presented distinct mechanical properties, with Eden having greater Storage modulus, Stiffness and Tan Delta, their recovery efficiency in percentage was similar for all mechanical properties. For example, they both lost around 20% from their respective Storage Modulus when wounded with a longitudinal incision on the stems. This can be explained by the fact that the parameters wound types and *Linum usitatissimum* cultivars are non-interdependent for all mechanical properties, thus both cultivars reacted the same way to wound types.

This stable efficiency rate between the two cultivars occurred despite the variability among plants in terms of intrinsic growth and reactions of stem tissues during wound healing represented by the significant random effect "samples". In contrast, plant structural responses (i.e. plant height and stem diameter) to mechanical damage were associated with more variability. The random factor "pot" representing the variability of environmental growth conditions was significant only for plant height and stem diameter. In addition, the parameters wound types and *Linum usitatissimum* cultivars were also interdependent. It indicates that the structural traits of Drakkar and Eden might be more sensitive to growth conditions than mechanical properties and wound healing. The strong background selection of the cultivars focused on fibre quality could explain our findings. However, plant height and stem diameter could be important traits concerning the quantity of fibres produced and could decrease fibre yield in case of wounded stems.

### Implication for technical self-healing material and concepts for composite materials

In addition to the use of plant fibres in composites for natural fibre-reinforced composites, plants are also a source for biomimetic self-healing materials [8, 9, 40]. Successful concepts developed in recent years were inspired from biological system such as blood flow vascular networks [41] and more recently from the vascular plant system of xylem and phloem [40]. These bioinspired materials include a segregated vasculature into fibre-reinforced polymer composite. The refilling property of the integrated vascular networks, thus allow repeated self-healing function [42].

In contrast to the concept of micro-tubes delivering a healing agent in the matrix, our findings with the two *Linum usitatissimum* cultivars showed that the matrix played an active role in the healing process: the healing occurred via the bast fibres (sclerenchyma) but also in other tissues such as vascular system (e.g. xylem) and the matrix (e.g. pith and cortical parenchyma). The stem structure was subjected to a profound reorganisation locally around the wound.

Most of the self-repair models for fibre-reinforced materials were inspired by hardwood species [40]. The *Linum usitatissimum* cultivars used during our investigation are annual plants presenting a short life history i.e. growing actively over a short period of time compared to a species such as oak (*Quercus robur* L.). Therefore, they do not experience the same constraints for their structure and have to constantly adjust to any event occurring during the rapid organ development such as wound healing. Furthermore, seed production is crucial for annual plants, consequently the structure bearing the seeds is of upmost importance, making healing of the stem decisive for the reproduction strategy. Therefore, wound healing in annual plant stems might confer another source for bioinspired materials by providing a dynamic approach of self-healing.

## Conclusion

This study demonstrated the possibility to use Dynamic Mechanical Analysis (DMA), coupled to more traditional plant observation techniques, to assess and compare the initial and healed properties of plant stems. Alongside with the better understanding of wound healing in plants and their implications in terms of mechanical and structural recovery, our findings show also the possible impact on textile quality and fibre yield. This study was performed during a young phase of the *Linum usitatissimum* cv. Drakkar and cv. Eden growth, when plants were building their stem and fibres. It would be interesting to evaluate the influence of damage that occurred during a later phase of development towards the end of the fibres building, i.e. when the structure is almost in its final shape. Another interesting aspect would be to investigate the responses with lignin deficiency *Linum usitatissimum* stems. Lignin being essential to wound healing, how would the plant cope in terms of physiology against pathogens and in terms of mechanical reinforcement? Given the increasing interest in self-healing materials and sustainable materials, further investigations of *Linum usitatissimum* plant fibres might be of particular interest. This famous plant represents also an invaluable source for self-healing material concepts as an invaluable point of departure for future applications for constructing technical self-healing fibre-reinforced composites.

## Supporting information

S1 Fig Full models and final models obtained after the model building approach for each parameter. Only significant terms were retained in final models.
(PDF)

S1 Table Raw data. (XLS)
