# Supplementary material for: Effect of mechanical damage and wound healing on the viscoelastic properties of stems of flax cultivars (Linum usitatissimum L. cv. Eden and cv. Drakkar)
Source: PLoS One. 2017 Oct 5;12(10):e0185958. doi: 10.1371/journal.pone.0185958 (PMC5628913; doi:10.1371/journal.pone.0185958)
Supplement: S1 Fig — Only significant terms were retained in final models. (PDF) [file pone.0185958.s001.pdf]

S1 Fig. Full models and final models obtained after the model building approach for each parameter. Only significant terms were retained in final models.

Legends:

```
LogSM is Log(storage modulus)
LogSTF is Log(stiffness)
LogSTRAIN is Log(strain)
type is type of wound
cv is cultivar of Linum usitatissimum
sample is individual
```

1 - Full models used to start the model-building approach:

(a) Storage Modulus

```
LogSM~LogSTRAIN*type*cv+(LogSTRAIN*type*cv|pot/sample)
```

(b) Stiffness

```
LogSTF~LogSTRAIN*type*cv+(LogSTRAIN*type*cv|pot/sample)
```

(c) Tan Delta

```
tan delta~strain*type*cv+(strain*type*cv|pot/sample)
```

(d) Plant Height at 95 DAS

```
H_95DAS~type*cv+(1|pot/sample))
```

(e) Plant Diameter at 95 DAS

```
diam~type*cv+(1|pot/sample)
```

2 - Final models retained after both random and fixed effects checking:

(a) Storage Modulus

```
LogSM~LogSTRAIN+type+cv+LogSTRAIN:type + LogSTRAIN:cv+(LogSTRAIN|sample)
```

(b) Stiffness

```
LogSTF~LogSTRAIN+type+cv+LogSTRAIN:type + LogSTRAIN:cv+(LogSTRAIN|sample)
```

(c) Tan Delta

```
lmer(tan delta~strain+type+cv+strain:cv+(strain|sample)
```

(d) Plant Height at 95 DAS

```
H_95DAS~type*cv+(1|pot/sample))
```

(e) Plant Diameter at 95 DAS

```
diam~type*cv+(1|pot/sample)
```
